# Sieve Plate Pores in the Phloem and the Unknowns of Their Formation

**DOI:** 10.3390/plants8020025

**Published:** 2019-01-22

**Authors:** Lothar Kalmbach, Ykä Helariutta

**Affiliations:** 1 The Sainsbury Laboratory, University of Cambridge, Bateman Street, Cambridge CB2 1LR, UK; 2 Institute of Biotechnology, University of Helsinki, 00014 Helsinki, Finland

**Keywords:** plant vasculature, phloem, development, sieve element, sieve plate, cell wall, plasmodesmata, *Arabidopsis*

## Abstract

Sieve pores of the sieve plates connect neighboring sieve elements to form the conducting sieve tubes of the phloem. Sieve pores are critical for phloem function. From the 1950s onwards, when electron microscopes became increasingly available, the study of their formation had been a pillar of phloem research. More recent work on sieve elements instead has largely focused on sieve tube hydraulics, phylogeny, and eco-physiology. Additionally, advanced molecular and genetic tools available for the model species *Arabidopsis thaliana* helped decipher several key regulatory mechanisms of early phloem development. Yet, the downstream differentiation processes which form the conductive sieve tube are still largely unknown, and our understanding of sieve pore formation has only moderately progressed. Here, we summarize our current knowledge on sieve pore formation and present relevant recent advances in related fields such as sieve element evolution, physiology, and plasmodesmata formation.

## 1. Introduction

**Figure 1:**
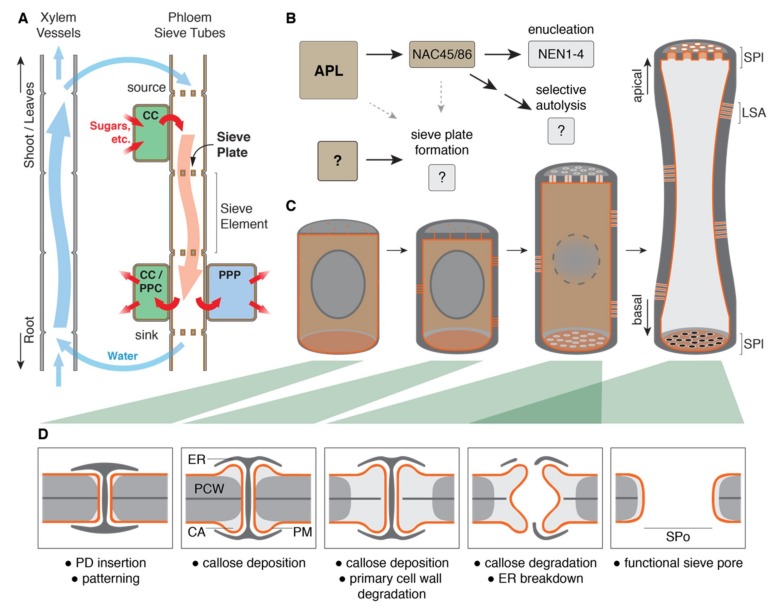
Sieve element differentiation and formation of the terminal sieve pores. (**A**) A schematic overview of the plant vascular system: Water and nutrients are transported from root to shoot and leaves through the xylem. In source leaves, loading of sugars from companion cells into the sieve elements increases the osmotic potential and generates pressure to push phloem sap towards sink tissues. There, sugars are unloaded through companion cells and possibly phloem parenchyma cells [12] or in the root through the phloem pole pericycle for further transport into adjacent cells [13]. (**B**) ALTERED PHLOEM DEVELOPMENT (APL) initiates sieve element differentiation through downstream transcription factors and enzymes which then control cellular remodeling processes like enucleation and selective autolysis. Sieve plate formation is influenced by APL and NAC DOMAIN CONTAINING PROTEIN (NAC) 45/86 but occurs also in the absence of these transcription factors. (**C**) The differentiation of an idealized sieve element: Cells are initially indistinguishable from their neighbors, yet the pattern of the future pores is already determined by apical and basal plasmodesmata. During further differentiation, callose is deposited around terminal plasmodesmata and lateral sieve areas are formed, while nuclear breakdown and cytoplasmic clearing begin. Once selective autolysis is completed, residual organelles (not shown) remain tethered to the lateral plasma membrane. Sieve pores are opened, and sieve elements stretch with the growing surrounding tissues. (**D**) The progression of the sieve pore formation: A plasmodesma with the protruding ER desmotubule is gradually transformed. Callose is deposited as cones around the plasmodesma, creating a callose plug, while cellulose of the primary cell wall is simultaneously degraded. Just until pore opening, callose maintains the cell wall integrity. Pore opening begins from the center of the structure and degrades the callose plug, including the remaining middle lamella, while autolysis removes the desmotubule. The size of the final pore is determined by the original extent of the callose plug. **CC**: companion cell; **PPC**: phloem parenchyma cells; **PPP**: phloem pole pericycle; **SPl**: sieve plate; **LSA**: lateral sieve area; **PD**: plasmodesma; **ER**: ER desmotubule; **PCW**: primary cell wall with middle lamella; **CA**: callose; **PM**: plasma membrane; and **SPo**: sieve pore.

Studying the plant vascular system has attracted considerable attention over the past decades. The vasculature enables apoplastic water and nutrient transport from the root to the shoot through root pressure and transpiration-powered transport through the xylem vessels. At the same time, the phloem transports various substances and assimilates, such as sugars, amino acids, lipids, proteins, RNAs, and hormones but also micronutrients and water, symplastically from photosynthetically active source tissues to photosynthetically inactive sink tissues [1]. The phloem thus distributes energy, molecular building blocks, and systemic signals for growth and development throughout the plant (Figure 1A). Phloem transport occurs through the sieve tube, a supracellular conduit consisting of the individual sieve elements, which are connected to each other through sieve pores in their end walls, a structure commonly referred to as sieve plates [2]. 

Most of our knowledge of molecular and genetic mechanisms of vascular development originates from work on the model plant species *Arabidopsis thaliana*. Xylem and phloem are specified and maintained from embryogenesis on through the concerted and mutually inhibiting action of auxin along the prospective xylem axis and cytokinin around the prospective phloem poles [3]. Initiated by a suite of tissue-specific as well as more broadly expressed factors, the protophloem sieve elements in *Arabidopsis* differentiate and become conductive within 20–25 cells away from the quiescent centre [4,5]. The protophloem sieve element is the first tissue to differentiate in the root meristem, a phenomenon which was recently reported to be controlled through a tissue-specific regulation of auxin transport and signalling in the early sieve element cell file [6]. Mechanisms of vascular patterning and early phloem development have been intensely studied and discussed over the recent years [2,3,4,5,7,8]. For a detailed overview of the topic, we refer the reader to the excellent work of our colleagues.

Most known genes involved in protophloem specification and development are present very early in the sieve element cell file, and their corresponding mutants display either a complete or stochastic absence of sieve element differentiation [5,7]. This suggests a role rather in the orchestration of the differentiation program than in the specific aspects of its actual execution. In *Arabidopsis*, sieve element differentiation is controlled by the Myeloblastosis-related (MYB) transcription factor ALTERED PHLOEM DEVELOPMENT (APL), which induces further downstream transcription factors and enzymes to manifest sieve element differentiation, such as the transcription factors NAC DOMAIN CONTAINING PROTEIN (NAC) 45 and NAC86 to induce nuclear breakdown [9,10] (Figure 1B). Sieve elements represent an extreme case of cellular differentiation and undergo cell wall thickening, enucleation, selective autolysis, and formation of the sieve plate (Figure 1C). While we have begun to decipher the process of enucleation [4,10], other processes during sieve element differentiation are far from being understood. For example, although continuous cell wall thickening has proved to be a dependable readout to characterize many early phloem development mutants in *Arabidopsis* [7], its regulation in the context of sieve element differentiation is poorly understood. 

The formation of sieve pores is critical to phloem function, and the accompanying biochemical changes in the sieve plate cell wall have been described already decades ago. Yet, virtually nothing is known about the involved cellular mechanisms, and previously described mutants in phloem differentiation reveal only little about the sieve plate. The double mutant *nac45/86* still forms sieve plates, suggesting that enucleation and sieve pore formation are controlled independently [10]. And even though the *apl* mutant displays profound alterations in protophloem sieve element formation, such as lignification of the cell wall comparable to xylem vessels, it still forms sieve plates [11].

In this review, we present functional aspects of sieve plates, examine the morphological changes during sieve pore formation, describe recent advances in plasmodesmata research, which are relevant to our understanding of sieve pores, and outline the necessary cellular processes for successful pore formation. We propose that in order to decipher the formation and adaptability of sieve plates, we need to consider the individual sieve pore as a tissue-specific microstructure, which depends on a tightly regulated interplay between the plasma membrane, the cell wall, and proteins.

## 2. Sieve Pores in the Sieve Plates of Angiosperms

The sieve pores in the apical and basal cell walls or end walls of the sieve elements were first described in various tree species some 180 years ago by the German forestry biologist Theodor Hartig [14]. Collectively, the apical and basal cell walls with their large pores are referred to as “sieve plates”. Most investigations on these sieve plates were descriptive in nature and studied their variable anatomy, their physiological importance with respect to vascular hydraulics, and their development. Perforations of these end walls of conducting cells are found in all vascular plants. Although not covered in this review, it is worth noting that sieve elements with terminal sieve pores have also independently evolved in the brown algae of the order *Laminariales*, which do not share a common multicellular ancestor with today’s land plants [15,16]. Sieve pores can be rapidly closed through the deposition of callose. In angiosperms, sieve pore occlusion has also been attributed to agglomerations of structural P-proteins in the sieve elements. This, however, has lately been questioned. P-proteins do not block translocation through sieve pores and their characteristics were recently reviewed [17]. 

In opposition to the pores in the apical and basal cell walls, perforations of the side walls are generally referred to as “lateral sieve areas” yet do not occur in all sieve elements [18]. In angiosperms, pores in the lateral sieve areas are generally of similar size across species and smaller than the pores in the sieve plates [19], with the exception of *Austrobaileyaceae*, a monotypic family found solely in the tropics of Queensland, Australia [20]. Gymnosperms, on the other hand, have so-called sieve cells, in which lateral and end walls do not substantially differ structurally. Here, all pores are of roughly the same diameter and generally considerably smaller than in angiosperms [21]. Sieve element end walls in angiosperms may be organized as simple sieve plates, where sieve pores are equally dispersed and are generally bigger, or as compound sieve plates, in which the pores are generally smaller and aggregated into several areas [19]. Additionally, sieve plates may be rather perpendicular to the lateral cell walls or inclined with the latter occupying a larger area and having generally smaller pores [19].

## 3. Sieve Plates Vary in Size, Pore Diameter, and Overall Architecture

Diameter and number of sieve pores in the sieve plate vary greatly between different species. Particularly large pores with diameters above 10 µm were observed in the gourds (*Cucurbita* sp., 10.3 µm), the genus *Tetracera* (13.1 µm), and the Tree of Heaven (*Ailanthus altissima*, 14.3 µm) while many other species have pores of around or less than 1 µm [19]. Pore size, sieve plate diameter, and length of the individual sieve element determine the conductivity of the sieve tube. As an approximation, the Hagen–Poiseuille equation for laminar flow can be used to estimate the hydraulic resistance *R* through a cylinder, which is inversely proportional to the radius to the power of four (*R ∝* 1*/r*^4^). Adaptations and refinements of the Hagen–Poiseuille equation to the case of the sieve tube demonstrate that the anatomy of the sieve plates is a key contributor to the overall hydraulic resistance [22], contributing around or more than 50% of the overall sieve tube hydraulic resistance [23]. For example, sieve plates and individual sieve pores in *Cucurbita* are approximately ten times larger than the ones found in the model plant *Arabidopsis thaliana*, which results in an approximately 100 times higher hydraulic sieve tube conductivity in the former. Yet, the effect of the sieve plate anatomy on overall phloem flow is more complex, and a few relatively large pores in the center of the *Cucurbita* sieve plates contribute most to overall sieve plate conductivity, while the smaller lateral pores are hydraulically much less relevant [24]. 

These hydraulic implications were already noted by Esau and Cheadle in the late 1950s, who proposed that because of the impact of the sieve plate on overall hydraulic resistance, plants likely evolved towards simpler sieve plates, which were found to generally have bigger pores [19]. However, two recent studies on the morphological features of sieve elements and sieve plates failed to identify such an evolutionary trend towards a specific sieve plate type. No correlation between the hydraulic conductivity and the organization of sieve pores in simple or compound sieve plates could be found. Instead, both types of sieve plates were found across the phylogenetic tree, and overall conductivity of the sieve tube correlated with the height of a tree and not with specific pore arrangements [25,26]. In fact, sieve plate morphologies appear to be much more variable than originally assumed. In terms of impacting hydraulic conductivity across the sieve plate, pore size is far more important than pore number. Indeed, sieve pore and tube diameters increase towards the base of a plant to increase conductivity [27]. This developmental plasticity in sieve pore diameters within individuals has also been observed in several tree species, allowing them to adjust hydraulic conductivity as they grow to avoid generating impossibly high phloem turgor pressures [26]. It is noteworthy that in gymnosperms, where sieve element and pore diameters do not increase with tree height, longer sieve cells and higher numbers of sieve areas are reported [25]. Such adaptations should also decrease overall hydraulic resistance, however to a lesser degree than the sieve pore widening observed in tall angiosperms. This is in accordance with generally lower transport speeds in gymnosperms compared to angiosperms [28].

## 4. Sieve Pore Formation during Phloem Development

Sieve pores differentiate from plasmodesmata and, once fully formed, continue to share several features with them. Plasmodesmata are small membrane-lined symplastic connections between neighboring cells. They contain a central endoplasmic reticulum (ER) connection called the desmotubule. The space between the desmotubule and plasma membrane is referred to as the cytoplasmic sleeve and allows intercellular cytosolic exchange [29].

Diameters of plasmodesmata range from 20–50 nm [30], while mature sieve pores are often in the micrometer range [19,24]. For example, assuming a final sieve pore in *Cucurbita* of approximately 5 µm necessitates a 100–250 times increase in diameter compared to the original plasmodesma structure, even the rather small sieve pores in *Arabidopsis* are still between 10–25 times as big as the preceding plasmodesmata. Enlarging an existing plasmodesma to a fully formed sieve pore, therefore, requires precisely timed and spatially controlled cell wall remodeling (Figure 1D).

The initial patterning of the end wall through the insertion of plasmodesmata can be considered the first step in sieve plate differentiation. Plasmodesmata can be rapidly closed through callose, a linear beta-1,3-glucan which is deposited around their necks, thereby constricting their aperture and effectively closing it [30]. The beginning of the transformation of a plasmodesma into a sieve pore appears similar. Callose is deposited around the edges to form a little cone, which protrudes slightly into the sieve element protoplast [31]. Continued callose deposition replaces cellulose in the primary cell wall, which is being progressively degraded around the plasmodesma. The middle lamella resists hydrolysis and disappears just before pore opening [31]. Eventually, the entirety of the future pore is filled with a callose plug, which maintains the cell wall integrity until pore opening [31]. Callose degradation commences at the middle lamella in the center of the pore and rapidly hydrolyzes the callose plug until only residual callose remains around the open pore. Despite the considerable variation in sieve plate architecture and pore sizes, this progression appears conserved at least within the dicotyledons [31,32].

### 4.1. Lessons from Plasmodesmata

Because of the close relation between plasmodesmata and sieve pores, it is helpful to understand plasmodesmata as such in order to understand their modification into sieve pores. Primary plasmodesmata are inserted into the cell wall during cytokinesis when individual ER strands are trapped between secretory vesicles, which fuse at the phragmoplast to give rise to the cell plate [33]. Secondary plasmodesmata instead are inserted post-cytokinetically. Since the plasmodesmata density hardly changes during cell elongation, secondary plasmodesmata are thought to be inserted predominantly into extending cell walls [34]. Simple plasmodesmata have only one single cytoplasmic connection but can mature into complex plasmodesmata, which are branched or funnel-shaped [29]. Recently, the additional distinction between type I plasmodesmata, which have a very narrow cytoplasmic sleeve, and type II plasmodesmata with nanoscale spokes between the ER desmotubule and the plasma membrane was introduced [35]. Plasmodesmata have attracted increasing attention for their various roles in plant development [36], plant–pathogen interaction [37], and phloem physiology [38,39], and their characteristic ultrastructure has recently been reviewed [40].

The plasma membrane of plasmodesmata is a distinct micro-domain enriched in sterols and sphingolipids and is characterized by a specific set of proteins [41]. Sterols and sphingolipids can make up to 30% and 10%, respectively, of all lipids in plant cells and are, therefore, often considered to be structural components of lipidic membranes rather than, for example, phosphatidylinositol phosphates (PIP) which occur in very low absolute concentrations but may act as specific membrane landmarks for intracellular trafficking [42]. Both, sterols and sphingolipids have been previously implicated in trafficking to the plasma membrane. For example, the *cyclopropylsterol isomerase 1* (*cpi1*) mutant lacks most sterols typically occurring in wild-type plants. This results in PIN-FORMED 2 (PIN2) polarity loss due to endocytic defects during cytokinesis and suggests a distinct role for sterols in defining cell polarity just after the establishment of the cell plate [43]. Intracellular dynamics of auxin transporters, in particularly the PIN proteins, are well studied and their fluorescent protein fusions are often used as markers for specific plasma membrane domains to elucidate subcellular trafficking towards these target membranes [44].

Biosynthesis of the sphingolipid ceramide can be inhibited using the synthesis inhibitor fumonisin B1 (FB1). This inhibition causes changes in overall lipid composition very similar to hypomorphic, non-lethal mutations in the ceramide biosynthesis genes *LONGEVITIY ASSURANCE ONE HOMOLOG 1,2,3* (*LOH1,2,3*). FB1 causes defects, which appear similar to treatment with the recycling inhibitor brefeldin A (BFA) and prevents proper localization of the auxin carrier AUXIN RESISTANT 1 (AUX1) and PIN1 due to interference with the biogenesis of the trans-Golgi network (TGN)/early endosomes [45].

### 4.2. The cher1 Mutant Connects Plasmodesmata Maturation and Sieve Pore Morphogenesis

A clear mechanistic connection between local enrichment in specific lipids and the maturation of plasmodesmata, and by extension sieve pores, is still missing. Yet, there is increasing evidence for membrane nano-domains enriched in specific lipids (also referred to as “lipid rafts”) around plasmodesmata, which are crucial to their formation and function [46]. Significant insights on linking plasmodesmata and sieve pore maturation to lipid homeostasis originate from the discovery of the *choline transporter-like 1* (*cher1*) mutants through various forward and reverse genetic approaches. *CHER1* is also known as *CTL1*, yet since *CTL1* originally described the unrelated enzyme *CHITINASE LIKE 1* [47], the name *CHER1* should be preferred.

The mutant *cher1* was initially identified in a screen for plants affected in vascular development. Sieve pores in *cher1* are less numerous and many remained occluded by the ER, which was not properly removed from the sieve pore aperture. This resulted in impaired translocation and unloading of cytosolic GFP, expressed from the promoter of *SUCROSE TRANSPORTER 2* (*SUC2::GFP*), which is routinely used as a marker for symplastic phloem transport. Additionally, *cher1* displayed changes in the amounts of choline and phosphocholine, which are precursors for the major plasma membrane lipid phosphatidylcholine. CHER1 localizes to the TGN/early endosome, the cell plate in dividing cells, and the developing sieve plate in sieve elements. Intriguingly, CHER1 only transiently localized to newly forming cell walls during cytokinesis but remained at the sieve plate—a localization which, however, was dependent on continued recycling as demonstrated by a treatment with BFA, which caused CHER1-YFP aggregation into larger intracellular structures, known as BFA bodies [48].

An independent approach made use of a ubiquitously expressed viral movement protein fused to a GFP to specifically label complex plasmodesmata (*35S::MP17-GFP*). *cher1* was identified from a mutagenized population screened for the lack of a GFP signal and hence reduction or absence of complex plasmodesmata. Although *cher1* had detectable changes in gross lipid composition, they did not appear dramatic. Still, *cher1* mutants showed a considerable reduction in *de novo* formed secondary plasmodesmata [49]. Additionally, proteomic analysis showed that *cher1* had a significantly reduced abundance of plasmodesmata proteins [50]. This could suggest that the mild overall lipid defects in *cher1* may in fact simply mask the subcellular complexities during the plasmodesmata formation and recruitment of plasmodesmata-specific proteins.

The *cher1* mutant also showed defects in the post-Golgi secretion of vesicles to the plasma membrane, a defect which had been attributed to changes in the lipid homeostasis. This resulted in a reduced abundance of several auxin transporters, consequently explaining various developmental defects of the *cher1* mutant as auxin-defects [51]. It will, therefore, be crucial to untangle the pleiotropic auxin-related defects of the mutant from the role of CHER1 during plasmodesmata and early sieve pore formation.

### 4.3. Origin of the Pore-Forming Plasmodesmata

It may appear a bit blurry as to whether primary or secondary plasmodesmata give rise to the sieve pores. Early electron-microscopy studies could not clarify this [32], more recent studies do not appear to have addressed this early aspect of sieve pore morphogenesis, and reviews of the field refer to the process generally as a restructuring of plasmodesmata without discussing their possible origin [2,39]. Since the sieve plate does not extend during differentiation, it has been assumed that the forming sieve plate is dotted by plasmodesmata from its very inception during cytokinesis and, hence, that sieve pores originate from primary, simple plasmodesmata [31]. This appears in contrast to the sieve pore defects in *cher1*, a mutant which has been reported to be specifically affected in secondary plasmodesmata [49]. It is imaginable, however, that the pore-forming plasmodesmata in *cher1* as such are unaffected primary plasmodesmata but that their maturation into sieve pores relies on trafficking events to the sieve plate, which are necessary for these pre-existing plasmodesmata to progress into sieve pores.

### 4.4. Callose Deposition Replaces Primary Cell Wall

Differentiation from a plasmodesma into a sieve pore progresses through the transient state of cellulose replacement with callose. This step maintains the cell wall integrity while simultaneously delimiting the size of the final pore [31]. It requires the correct localization and activity of cell wall degrading enzymes such as cellulases and pectinases, which the precise identity of, however, has not been determined [29]. Cell wall modifying enzymes belong to notoriously large protein families. For example, the *Arabidopsis* genome encodes for the staggering number of 379 glycosyl hydrolases, of which more than 100 catalyze the breakdown of pectins [52]. There is presently no functional data for by far most of these enzymes and very little indications which of these may be involved in the specific task of sieve pore formation.

Callose deposition, in general but also in sieve plates, is presently better understood than cellulose degradation. The *Arabidopsis* genome encodes for twelve callose synthases, for which two independent nomenclatures exist. They are either referred to as *CALLOSE SYNTHASE* (*CalS*) *1* to *12* [53], which will be used here, or *GLUCAN SYNTHASE-LIKE* (*GSL*) *1* to *12* [54]. Besides around plasmodesmata and the sieve plates, callose is transiently deposited in the cell plate, where it is replaced once cytokinesis is completed [53,55]; it is deposited in growing pollen tubes [56], and around the base of trichomes [57]. Callose is also rapidly deposited upon physical damage of plant cells or pathogen infection [58]. Comprehensive reviews on the different roles of callose synthesis and depositions in plants have been published previously and remain relevant [54,59,60]

*CalS7* (i.e., *GSL7*) was identified as the callose synthase to be predominantly expressed in the sieve elements, and the corresponding mutant *cals7* lacks all callose deposition in sieve tubes [61]. Although *cals7* mutants still make sieve pores, the final pores are generally smaller and lack the characteristic surrounding callose collar [62]. Mutants of *cals7* are a bit smaller than wild-type plants, yet their shorter root phenotypes can be largely complemented by the addition of sucrose to the growth medium [61]. This suggest, that even though callose is largely degraded during the course of sieve pore formation, its deposition is important for sieve tube function and carbon transport to the growing root tip. Accordingly, *cals7* mutants accumulate starch in source leaves but are carbon deprived in sink tissues [62]. Yet, it has been debated if sieve pores, once they are fully formed, still contain any callose at all and if any observed callose is in fact merely an artefact of the preparations and the rapidly induced callose deposition due to the inflicted trauma. When whole plants are rapidly fixed, no callose could be observed in mature sieve elements of the duckweed *Lemna minor*. This has, however, been contested by Esau and Thorsch, who argued that “*the phenomenon is so consistent and orderly in the differentiating sieve element that it helps to interpret the sequence of events (…)*” [31]. Although callose deposition in sieve elements of *Arabidopsis* occurs very fast, sieve plate callose is also observed in rapidly fixed tissues, suggesting that at least in *Arabidopsis*, the sieve plate contains callose also in a non-challenged state [61].

Trauma-induced callose deposition in the sieve elements of *Arabidopsis* occurs also in lateral cell walls. Intriguingly, this ubiquitous wound-induced callose is equally absent in the *cals7* mutant [61]. This may suggest that rather than being exclusive to the development of the sieve pores, CalS7 mediates callose deposition in sieve elements in general. Yet, depending on developmental and environmental cues and necessities, which vary over time, CalS7 may preferentially be active in different plasma membrane domains of the sieve element. When the *cals7* mutant is complemented with the dominant active *icals3m* allele of *CalS3* (i.e., *GSL12*) expressed from the phloem-specific *APL* promoter (*APL::icals3m*), callose deposition not only is overall excessive in the sieve tube but also appears preferentially at the sieve plates of differentiating root sieve elements [63]. Unfortunately, callose synthases have proved difficult to use as fluorescent protein fusions. While the expression domains of many *CalS* genes have been identified [64], their precise subcellular localizations and dynamics during callose depositions are still unknown, with the exception of CalS3 [63] and CalS12 (i.e., GSL5 or PMR4) [65].

### 4.5. Pore Opening

Rapid degradation of the callose plugs opens the sieve pores and makes the newly differentiated sieve element a fully functional member of the sieve tube. Pore opening has been reported to occur around the time of enucleation [32] or right after enucleation is completed [13]. This is indirectly supported by transcriptional analyses, which showed that some specific cell wall remodeling enzymes are NAC45/86-dependent while others are not. For example, sieve element callose deposition through CalS7 appears APL-dependent but NAC45/86-independent while some beta-1,3-glycosyl-hydrolyases that degrade callose are direct targets of NAC45/86 [10]. This may imply that callose degradation during sieve pore opening is at least partially under the same control mechanisms as enucleation but that the preparatory modifications of the cell wall are distinct events.

It may appear odd why plants first degrade parts of their primary cell wall to replace it with callose just to degrade the callose plugs shortly after. The chemical and mechano-physical properties of cellulose and callose are vastly different. Cellulose is organized in long fibrils and often occurs in a crystalline state, whereas callose is a linear, relatively shorter polymer, which is rather amorphous [66]. Callose is also softer than cellulose and its addition to a cellulosic matrix even as the minor polysaccharide reduces the stiffness at least in artificial cellulose–callose mixtures [67]. Therefore, callose is considered to be easier to hydrolyze than cellulose, thus allowing the plant a rapid collapse of the callose plugs to make a functional pore. Since callose deposition also occurs very rapidly, this process is reversible and can adapt quickly to external cues [61,63]. The relatively mild developmental phenotype of the *cals7* mutant suggests that this entire mechanism of callose deposition for subsequent rapid degradation is not essential for phloem function and plant survival. Yet, the rapid pore opening and closure it allows may have been exceptionally advantageous in eco-physiological and evolutionary terms, analogous to, for example, the Casparian strip of the endodermis: an evolutionarily highly conserved feature, which the integrity of is not absolutely crucial for development but highly relevant in a physiological context [68,69]. As previously mentioned, gymnosperms have considerably smaller pores than angiosperms [25]. Intriguingly, just like the *Arabidopsis cals7* mutant, not only conifers and cycads but also mosses and some ferns lack callose in their sieve cell end walls and only produce callose upon wounding [70,71,72]. It is, therefore, tempting to speculate that the successive deposition and degradation of callose, which occurs in angiosperms, may have been a key evolutionary invention to allow for larger sieve pores.

## 5. Conclusions and Perspectives

Conceptually, the formation of a sieve pore requires polar cargo delivery to or retention at the apical and basal pore-forming plasmodesmata. Secreted enzymes to degrade the cell wall, such as glucanases and pectinases, have to be properly secreted but kept in place to limit their hydrolytic activity to the forming sieve pore. This could be achieved either through interactions with integral plasma membrane proteins, which may act as scaffolds, or through direct attachment to the plasma membrane. Many secreted glucanases and pectinases are, in fact, anchored in the outer leaflet of the plasma membrane through glycosylphosphatidylinositol (GPI) anchors [73] and may be localized through a specific lipid microenvironment. Callose synthases would equally need to be localized to the forming pore, and indeed, CalS3 localizes preferentially to plasmodesmata [63]. CalS proteins are structurally similar, and other callose synthases may exhibit similar subcellular distributions.

Since so little is known about the molecular and genetic mechanisms of sieve pore formation, it is worth looking into other tissue-specific cell wall modifications for inspiration. Pit formation in the differentiating metaxylem depends on two mutually exclusive mechanisms of microtubule depolymerization and stabilization through membrane-to-microtubule tether proteins. The local activation of Rho of plant GTPase 11 (ROP11) recruits MICROTUBULE DEPLETION DOMAIN 1 (MDD1) and Kinesin-13A to locally depolymerize microtubules [74,75]. On the other hand, IQ67 DOMAIN PROTEIN 13 (IQD13) stabilizes microtubules around the active ROP11 domain, thus restricting cortical microtubule depolymerization [76]. Their combined action excludes microtubule-guided cellulose synthases during secondary cell wall formation specifically from a plasma membrane region which then gives rise to a pit. As another example, the formation of the lignified Casparian strip in the endodermis requires the local production of reactive oxygen species and the positioning of PEROXIDASE 64 (PER64) into a ring around the cell. The localization of the biosynthetic machinery is determined by the endodermis-specific CASPARIAN STRIP MEMBRANE DOMAIN PROTEIN 1 (CASP1) in the plasma membrane [77,78], which is secreted in a localized fashion towards a PI(4,5)P_2_ landmark in the plasma membrane [79]. This mechanism allows the translation of an unstable lipid motif in the cytosolic leaflet of the plasma membrane into a localized polymer deposition in the cell wall. In the protophloem, although subcellular spatial distributions are unknown, changes in overall PI(4,5)P_2_ levels have been reported to impede sieve element differentiation [80,81].

It is entirely speculative whether similar mechanisms may govern the formation of the sieve pores, but advances in tissue-specific transcriptomics will enable targeted mining for sieve element differentiation factors, and improvements in live cell imaging in resolution and sensitivity already are facilitating advanced *in vivo* investigations in deep-lying plant tissues. After the decade-long hiatus in studying sieve plate differentiation and sieve pore formation, it is time to look at them again.
